# Investigating the Links Between Vaccination Against COVID-19 and Public Attitudes Toward Protective Countermeasures: Implications for Public Health

**DOI:** 10.3389/fpubh.2021.702699

**Published:** 2021-07-21

**Authors:** Ruishi Si, Yumeng Yao, Xueqian Zhang, Qian Lu, Noshaba Aziz

**Affiliations:** ^1^School of Public Administration, Xi'an University of Architecture and Technology, Xi'an, China; ^2^College of Economics and Management, Northwest A & F University, Yangling, China; ^3^College of Economics and Management, Nanjing Agricultural University, Nanjing, China

**Keywords:** COVID-19, vaccination, protective countermeasures, PSM, China

## Abstract

The COVID-19 pandemic caused by the novel coronavirus, SARS-CoV-2, is spreading globally at an unprecedented rate. To protect the world against this devastating catastrophe, vaccines for SARS-CoV-2 have been produced following consistent clinical trials. However, the durability of a protective immune response due to vaccination has not been confirmed. Moreover, COVID-19 vaccination against SARS-CoV-2 is not 100% guaranteed, as new variants arise due to mutations. Consequently, health officials are pleading with the public to take extra precautions against the virus and continue wearing masks, wash hands, and observe physical distancing even after vaccination. The current research collected data from 4,540 participants (1,825 vaccinated and 2,715 not vaccinated) in China to analyze this phenomenon empirically. The propensity score matching (PSM) model is employed to analyze the impact of vaccination against COVID-19 on participants' attitudes toward protective countermeasures. The findings showed that gender, age, education level, occupation risk, individual health risk perception, public health risk perception, social responsibility, peer effect, and government supervision are the main drivers for participants to be vaccinated with COVID-19's vaccines. The results further show that vaccination lessened participants' frequency of hand washing by 1.75 times and their compliance frequency intensity of observing physical distancing by 1.24 times. However, the rate of mask-wearing did not reduce significantly, implying that China's main countermeasure of effective mask-wearing effectively controls COVID-19. Moreover, the findings indicate that a reduction in the frequency of hand washing and observing physical distance could cause a resurgence of COVID-19. In conclusion, factors leading to the eradication of SARS-CoV-2 from the world are complex to be achieved, so the exploration of COVID-19 vaccination and people's attitude toward protective countermeasures may provide insights for policymakers to encourage vaccinated people to follow protective health measures and help in completely defeating the COVID-19 from the globe.

## Introduction

The COVID-19 pandemic has caused devastating harm worldwide, affecting many industries and resulting in the most severe economic recession since World War II (1, 2). According to the statistics released by Johns Hopkins University in the United States (June 5, 2021), the COVID-19 pandemic has infected 170 million people and caused 3.7 million fatalities globally. The World Health Organization (WHO) explained how the COVID-19 new variants mutate and spread rapidly. One of the mutations in the sequence of the viral receptor-binding domain of the spike protein, N510Y, is believed to enhance the viral transmissibility, and infectivity by increasing the affinity of the viral spike protein to its receptor (3). The swift virus transmission and the accelerated growth in the number of cases compelled the urgent development of an accurate and effective vaccine. It is clear that vaccinations have transformed global health and have enormous life-saving potential in their ability to boost immunity against this contagious disease. Countries worldwide are devoting themselves to develop effective vaccines against COVID-19 to effectively control the pandemic. Unfortunately, a completely effective drug has yet to be developed. Meanwhile, epidemiologists believe COVID-19 can be curbed by implementing strict countermeasures such as wearing masks, hand washing, and maintaining physical distance (4). Therefore, high anticipations are being placed on protective countermeasures in the fight to control COVID-19 and, in turn, to prevent pandemic-induced fatalities in nations worldwide.

Existing epidemiological and experimental research demonstrates that the main source of COVID-19 infections is aerosols (5), which are generally poly-dispersed droplets and particles and have many different sizes (6, 7). Infected aerosols easily spread in confined spaces through daily activities (e.g., exhaling, talking, coughing, and sneezing) and medical procedures (e.g., tracheal intubation, non-invasive ventilation, bronchoscopy, and tracheotomy) (8, 9). Accordingly, epidemiological evidence has confirmed the essential role of ventilation in reducing the risk of people exposed to aerosol infections. In an enclosed space, the airborne viral concentration from an infected person will build up over time to a level that depends on the ratio of the emission rate (10) to the number of fresh-air exchanges per hour (11). In other words, The risk then depends on the duration of exposure as well as the fresh air ventilation rate (12). The ventilation intensity depends on the perceived intervention, and not all places have good ventilation conditions limited by poor economic and environmental conditions (13). The ventilation time is also relatively uncertain about evaluating accurately and scientifically (14). Therefore, it is relatively difficult to eliminate the risk of SARS-CoV-2 transmission through aerosols (15).

Over the past one and a half years, many studies have confirmed the effectiveness of health-protective measures against COVID-19 (16, 17). Before the development of vaccines, wearing masks was regarded as an essential public health measure to halt the transmission of COVID-19 (18, 19). Based on risk management, China has provided medical staff and the public with suggestions concerning using masks with different protection levels, thereby significantly controlling COVID-19 (20). Moreover, compulsory mask-wearing has resulted in a four-fold reduction in daily mortality and a 2% daily reduction in new cases in the United States (19, 21). Mouth and nose droplets from infected patients can easily transmit to other individuals (22). In this context, a recent research by Gharpure et al. (23) confirmed that frequent handwashing is a substantial measure in reducing the transmission intensity of the COVID-19 infection. Additionally, the droplets produced by coughing or sneezing have a 1.2–2.4 m transmission distance (22). Therefore, maintaining physical distance can further reduce the spread of SARS-CoV-2 from person to person (24). An analysis in Wuhan and some lockdown cities in Italy and Spain showed that negligence in maintaining physical distancing had measurable results. The epidemic in these cities quickly peaked (25). There is also credible evidence that a physical distance policy of at least 1 meter may significantly reduce the intensity of infections. As many recent researchers have established, a distance of 2 m may be even more effective (26). However, scholars are aware that physical distancing is not a viable long-term countermeasure in relationship networks (26, 27). Wide-scale immunization and people's voluntary uptake of vaccines are what allows them to live normal lives if the immunization programs are successful (24, 28, 29). The COVID-19 vaccine is seen as one of the requirements for the true and permanent “opening up” of societies worldwide.

On April 13, 2021, the WHO reports revealed that there are currently 235 vaccines under development, 63 of which have entered clinical trials (30). Although these vaccines utilize different development platforms, including classic and mature approaches using inactivated whole virions, live-attenuated, recombinant protein, and vectored vaccines, as well as promising novel vaccines such as the DNA and mRNA vaccines, the S-spike protein is seen as a crucial target of COVID-19 vaccine (31, 32). On July 22, 2020, China officially launched the COVID-19 vaccination and prioritized special groups such as medical staff. On December 31, 2020, the WHO announced the approval of Pfizer Biotech's COVID-19 vaccine, the first emergency use vaccine authorized by the WHO. As of March 31, 2021, major countries or regions severely affected by COVID-19, such as China, the United States, Brazil, India, Japan, and the European Union, have started to vaccinate their populations against COVID-19, aiming to achieve herd immunity by promoting individual immunity against SARS-CoV-2 (1, 33).

Meanwhile, the WHO chief scientist Sumia Swaminatan appealed to those vaccinated to continue to engage in protective health measures such as wearing a mask, handwashing, and keeping physical distance. This urgent message is generally accepted and endorsed globally, chiefly for the following reasons. First, vaccine hesitancy is rising globally, and herd immunity has not yet been achieved (34). Quite a few people are afraid of and reluctant to get the COVID-19 vaccination. Latkin et al. (35) used a socio-ecological framework to explore Americans' intentions regarding the COVID-19 vaccination. The results found that only 59.1% of people intended to get the vaccination. Based on a cross-sectional research of 3,261 adults, Paul et al. (36) reported that 16% of the respondents displayed high levels of mistrust about vaccines, 14% of respondents reported their unwillingness to get the COVID-19 vaccination, while 23% were unsure. The main reason was that some vaccines had only been authorized for urgent use after their phase II clinical trials (37). Scientific experiments still need to establish whether adverse side effects such as fever, thrombosis, and death, have a causal relationship with the COVID-19 vaccines (38, 39). The AstraZeneca and the Johnson & Johnson vaccines have been abandoned in some countries because of adverse side effects such as thrombosis. Second, the effective protection period of the COVID-19 vaccines is uncertain. Not all animal models perfectly mimic human COVID-19 infection and immune responses (40). Moreover, the longest established protection period for the existing scientifically verified vaccines is only 1 year. Millions of people have been vaccinated with multiple types of vaccines, and the level of antibodies that can effectively neutralize SARS-CoV-2 requires long-term evaluation and monitoring (39). Third, the continued mutation of SARS-CoV-2 has posed severe challenges to the protective efficacy of existing vaccines. By June 15, 2021, the WHO had been officially notified about mutations of SARS-CoV-2 since its emergence. The variants of concern are mainly related to the B.1.1.7 mutation in United Kingdom (Alpha variant), the B.1.351 mutation in South Africa (Beta variant), the P.1 mutation in Brazil (Gamma variant), and the B.1.617.2 mutation in India (Delta variant) (41, 42). Epsilon, Zeta, Eta, Theta, Iota, Kappa, and Lambda are variants of interest named by the WHO.

Although some countries such as China, France, and United States believe that, in general, the mutations of SARS-CoV-2 have not had a detrimental impact on related treatments, drugs, and vaccines, the future risk is still uncertain, and it is a matter of extreme urgency to design more targeted and effective vaccines (43). Lastly, the age for vaccination is generally accepted to be 18 years and older as determined by clinical trials. The participants' physical condition is strictly screened to exclude people younger than 18, and those who are unsuitable for vaccination in China (40, 44). However, in the USA, Pfizer–BioNTech mRNA vaccine clinical trials for children under age 12 are ongoing, and people between ages 13–16 are being vaccinated, and protection is 100%(45). The Australian health authorities recommend the vaccine for anyone 16 years old and over. Additionally, the global distribution of COVID-19 vaccines is not completely fair, especially since developing countries are unable to purchase enough vaccines (46). It can be inferred that there is still a long way to go before worldwide herd immunity is achieved (47). Therefore, although people are being vaccinated, they still need to engage in strict health-protective measures to reduce possible risks in the future.

As of March 31, 2021, in China, five COVID-19 vaccines had been approved for conditional marketing, and the number of vaccinated people reached 170 million. Although COVID-19 vaccines are free and optional in China, the above analysis indicates that vaccine hesitancy, the uncertain protection period, SARS-CoV-2 mutations, and the limited vaccination population pose several challenges for vaccine effectiveness. Consequently, the government has always asked vaccinated and non-vaccinated people to observe health-protective measures such as wearing masks, handwashing, and keeping physical distance. In the current research, we used online platforms in China to recruit 4,540 participants, and we used the propensity score matching (PSM) model to empirically analyze the impact of the COVID-19 vaccination on vaccinated participants' health-protective measures and to further discuss whether participants' protective measures had changed after vaccination. To our knowledge, no other research has examined the impact of the vaccination against COVID-19 on attitudes of people toward protective health measures. It is of crucial importance to understand the factors affecting behavior after COVID-19 vaccination. Vaccinated individuals may represent the most realistic focus of public health communication programs encouraging the continuation of the same countermeasures even after vaccination. As vaccinated individuals begin to constitute a more significant number within the population, maintaining their health-protection measures is paramount. Consequently, there is an urgent need for a more updated and nuanced understanding of attitudes toward protective countermeasures even after vaccination to provide tailored health advice for the public. The findings of this research have potential significance in helping policymakers identify and adapt interventions that increase the implementation of strict countermeasures even after vaccination. It is crucial for public health that such strategies are implemented and rolled out to maximize adherence to the measures among the general population.

The structure of the rest of the paper is as follows. The methodology section presents the data sources and the analytical strategies. Then, the estimated results are set out in the Results and Discussion section. We conclude with possible policy recommendations.

## Materials and Methods

### Data Collection

The data presented in this research were collected from vaccinated and non-vaccinated individuals from the Zhejiang, Hubei, and Shaanxi provinces of China from March 1st to 21st, 2021. These provinces were selected because they represent China's eastern, central, and western economic developments. The vaccine administered in these provinces is SARS-CoV-2 vaccine (Vero Cell) manufactured by Sinovac Life Sciences Co., Ltd. This vaccine is administered in two doses 2–4 weeks apart for people over 18. The data were collected from vaccinated and non-vaccinated individuals. Only those who had received two doses were qualified to complete the questionnaire, and they were asked to upload their vaccination certificates (48). After discarding the 285 blank or invalid questionnaires, we had 4,540 valid questionnaires out of 4,825, a questionnaire efficiency of 94.09%. In the sample, 1,825 participants had been vaccinated, and 2,715 participants had not been vaccinated. Moreover, we took occupation type as the exclusion and restriction criteria for participants. The survey data were not collected from health workers because their occupational requirements, risk awareness, and personal protective measures are likely to be much higher than those of the general population. The inclusion of health workers could have led to biased results. Most importantly, participants are anonymous during the data collection and processing. This research has obtained informed consent concerning the scientific use of data and guaranteed participants' privacy.

### Variable Selection

The variables included in the research were outcome, treatment, and covariates. The outcome variable is participants' health-protective measures, that is, wearing masks, handwashing, and keeping physical distance. Specifically, “the time spent per day wearing a mask in a public place” in the questionnaire represents wearing a mask, “the number of times of washing hands per day” represents handwashing, and “compliance intensity of keeping physical distancing of more than 1 meter (1 = very weak, 2 = weak, 3 = general, 4 = strong, 5 = very strong).” We selected the COVID-19 vaccination as the treatment variable; if the individual was vaccinated with the COVID-19 vaccine, the value was assigned as 1; if the individual was not vaccinated, the value was 0. Therefore, there were self-selection samples in the treatment variable. In line with related research conducted by Si et al. (49), we selected some other variables as covariates. The variables included gender, age, education level, individual health risk perception, public health risk perception, social responsibility, cultural roots, peer effect, government supervision, and accessibility to health-protection products.

We applied the independent sample *t*-test to analyze the differences in variables between the vaccinated and non-vaccinated individuals. Table 1 shows that the *t*-test results reject the null hypothesis and that there is no difference between the vaccinated participants in experimental group (A) and the non-vaccinated participants in control group (B). The results in Table 1 further reveal that compared with the non-vaccinated individuals, the number of handwashing times for the vaccinated individuals is reduced by 3.302, and the compliance intensity for keeping a physical distance of more than 1 meter was reduced by 1.817. However, there is no noticeable difference in the average time of wearing masks per day between the vaccinated and the non-vaccinated participants. Moreover, apart from cultural roots and accessibility to health-protection products, other covariates are also significantly different between the vaccinated and non-vaccinated individuals.

**Table 1:** Variables' differences between vaccinators and non-vaccinators.

**Variables**	**Definition and assignment**	**Vaccinators**	**Non-vaccinators**	**Differences**
		**(A)**	**(B)**	**(A–B)**
Wearing mask	Average time of wearing mask per day in the supermarket, etc. public place (hour)	3.752	3.924	−0.172
Handwashing	Number of times of washing hands per day (times)	4.651	7.953	−3.302^***^
Keeping physical distancing	Compliance intensity of keeping physical distancing more than 1 meter (1 = very weak, 5 = very strong)	2.085	3.902	−1.817^***^
Gender	Woman = 0, man = 1	5.016	4.805	0.211^*^
Age	Actual age (year)	49.205	43.280	5.925^***^
Education level	Education time (year)	14.205	11.602	2.603^***^
Individual health risk perception	The COVID-19 seriously threatens individual health. (1 = strongly disagreement, 5 = strongly agreement)	4.209	3.705	0.504^***^
Public health risk perception	The COVID-19 seriously threatens public health. (1 = strongly disagreement, 5 = strongly agreement)	4.392	3.806	0.586^***^
Social responsibility	Taking health protective measures is a social responsibility. (1 = strongly disagreement, 5 = strongly agreement)	4.175	3.608	0.567^*^
Cultural roots	Wearing mask etc. health protective measures is belonged to behavioral culture. (1 = strongly disagreement, 5 = strongly agreement)	3.605	3.610	−0.005
Peer effect	Taking health protective measures is affected by other behavior. (1 = strongly disagreement, 5 = strongly agreement)	4.025	3.042	0.983^***^
Government supervision	The intensity of government supervision of individual health protective measures (1 = very weak, 5 = very strong)	3.640	3.205	0.435^***^
Accessibility to health-protection products	It is easy to buy products such as masks. (1 = strongly disagreement, 5 = strongly agreement)	4.016	4.475	−0.459

*, **, *** *Represent the significance level of 10, 5, and 1%, respectively*.

Because vaccination is a voluntary “self-selection” behavior, the differences among some outcome variables cannot be attributed to the COVID-19 vaccination. In addition, they may be influenced by other covariates such as gender, age, education level, individual health risk perception, public health risk perception, social responsibility, peer effect, and government supervision. Therefore, we used PSM to explore the impact of the COVID-19 vaccine on participants' health-protective measures.

### Statistical Analysis

Compared with existing research methods, the reasons for using PSM to explore the impact of the vaccination against COVID-19 on participants' health-protective measures are as follows. First, the vaccination is based on the principle of voluntary action. Therefore, the division of vaccinated and non-vaccinated individuals in the sample is not random. Therefore, PSM is used to solve the problem of sample “self-selection” (50). Second, because the initial endowments of the treatment group (vaccinated) and the control group (non-vaccinated individuals) are different, there is an obvious “selection bias.” Therefore, PSM is applied to analyze the consistency of health-protective measures in the treatment group and the control group (51). Lastly, PSM can solve the “missing data” issue by constructing a counterfactual framework to observe the health-protective measures of vaccinated individuals in non-vaccination situations (52). The research steps of this paper are as follows:

The Logit model is employed to estimate the fitted value (the propensity score value) of the conditional probability of participants vaccinated.

(1) $$\begin{array}{r} PS_{m}=Pr\left[L_{m}=1|X_{m}\right]=E\left[L_{m}=0|X_{m}\right] \end{array}$$

where *L*_*m*_ = 1 means participants who have been vaccinated with the COVID-19 vaccine *L*_*m*_ = 0 indicates participants, who have not been vaccinated with the COVID-19 vaccine. *X*_*m*_ signifies an observable covariate, such as gender, age, education level, individual health risk perception, public health risk perception, social responsibility, peer effect, and government supervision.

The treatment group and the control group are matched. We selected three matching methods: K-nearest neighbor, caliper, and kernel matching. In particular, K-nearest neighbor matching is based on the value of PSM among the nearest K different groups of individuals. The K was set to 4, and one-to-four matching was performed to minimize the mean square error. Caliper matching refers to matching by restricting the absolute distance of the propensity score. We set the caliper to 0.020 to match observations with a 2% difference in propensity score values. Core matching refers to matching vaccinated participants by setting a propensity score of 0.060 on the broadband and weighted average of the control group samples in the broadband.

The difference in health-protective measures between the treatment and the control group was calculated by the average treated effect (ATT). Finally, we obtained the impact of the COVID-19 vaccination on participants' health-protective measures.

(2) $$\begin{array}{l} ATT=E\left(D_{1m}|L_{m}=1\right)-E\left(D_{0m}|L_{m}=1\right)\\ \;=E\left(D_{1m}-D_{0m}|L_{m}=1\right) \end{array}$$

where *D*_1*m*_ is the health-protective measures of participants vaccinated, *D*_0*m*_ is the protective health measures of participants vaccinated (assuming that they are not vaccinated), *E*(*D*_1*m*_|*L*_*m*_ = 1) can be directly observed, *E*(*D*_0*m*_|*L*_*m*_ = 1) cannot be directly observed, and it is a counterfactual result. Therefore, PSM is an appropriate approach to construct the corresponding substitute index.

Common support domain and balance tests were also conducted. The common support area test determines whether the control and treatment groups have a common support area and a large overlap in the value range. The balance test judges the matching quality by comparing significant differences in covariates between the treatment and the control groups.

## Results and Discussion

### Estimation of Participants Selecting the COVID-19 Vaccine

A matching environment with the highest similarity was created to screen suitable covariates. The Logit model was employed to estimate the selection equation for participants' vaccination intention to ensure PSM quality. Table 2 shows the estimated results of the model. The findings show that gender, age, education level, occupation risk, individual health risk perception, public health risk perception, social responsibility, peer effect, and government supervision can actively drive participants to be vaccinated with the COVID-19 vaccine. Specifically, due to the heterogeneity of perceived risk and fear of death, there was a marked gender difference in vaccine attitudes (53). Consistent with Chu's and Liu (33) related research, our research confirms the enthusiasm and initiative of men in the COVID-19 vaccination. However, previous studies have also reached the opposite conclusion, just as Latkin et al. (35) hold that females generally express greater intentions to obtain a COVID-19 vaccine than males. These findings further suggest that vaccination campaigns should consider gender differences in attitudes and acceptance. The elderly are the primary susceptible group. Statistical data from China, United States, and India also show a higher mortality rate among elderly COVID-19-infected persons (29, 54, 55). Consequently, the older the people are, the stronger they have the intention to be vaccinated. Many studies have reached a more consistent conclusion, that is, the higher the education level of people, the more scientific and comprehensive they will evaluate the safety, effectiveness and side effects of the COVID-19 vaccine. Eventually, they will respond to the government's call and actively vaccinate (48, 56). In our research, we innovatively divide health risks into individual health risks and public health risk perception. Consistent with Cohen and Rodgers (57) and Chen et al. (58) research results, in terms of the prevention and control of COVID-19, individual health risks and public health risk perception are interrelated and supported. Furthermore, the path from individual health risk perception to public health risk perception is mainly individual social responsibility (59). Moreover, formal social norms (government supervision) and informal social norms (peer effect) have become essential factors to lead people to vaccinate. Our research further confirms the views of Andrews et al. (60), who considered that individual public health behavior has strong externalities, and government supervision and peer effect are reasonable paths to realize the internalization of externalities.

**Table 2:** Estimation results of vaccination selection equation based on logit model.

**Variables**	**Selection of the COVID-19's vaccine**	
	**Coefficient**	**Standard error**
Gender	1.025^*^	0.563
Age	0.894^***^	0.344
Education level	0.626^**^	0.292
Individual health risk perception	0.902^***^	0.347
Public health risk perception	0.407^*^	0.226
Social responsibility	0.702^**^	0.319
Cultural roots	0.528	0.340
Peer effect	1.505^***^	0.501
Government supervision	1.024^***^	0.379
Accessibility to health-protection	0.305	0.195
products		

*, **, *** *Represent the significance level of 10, 5, and 1%, respectively*.

Cultural roots and accessibility to health-protection products have no significant influence on participants' vaccination intention. Cultural roots are the deepest driving force of individual behavior intention and decision (61, 62). Epidemic experience and environmental pollutions are key reasons people wear masks and are embedded in China's behavioral culture and social patterns (63, 64). Thus, cultural roots may conceal the impact of vaccination on people wearing masks and the limitation is discussed in research limitation part. As discussed above, many studies have also confirmed the importance of wearing masks and health-protective measures to prevent and control the spread of COVID-19 (65–67). Therefore, it is believed that under strict health-protective measures, the risk of exposure to SARS-CoV-2 is relatively low, and the time of COVID-19 vaccination can be delayed. Moreover, at the beginning of the COVID-19 outbreak, health-protective products such as masks became strategic materials for competition among countries, thereby underlining the significance and effectiveness of health-protective measures (57, 68). However, the current market supply of health-protective products is relatively sufficient, and participants' enthusiasm for vaccination is not as high as it should be. Consequently, cultural roots and the accessibility to health-protective products cannot drive participants to get vaccinated. Therefore, the current research excluded these variables before PSM to ensure the quality of matching.

### The Tests of Common Support Domain and Balance

#### Common Support Domain Test

**Figure 1:**
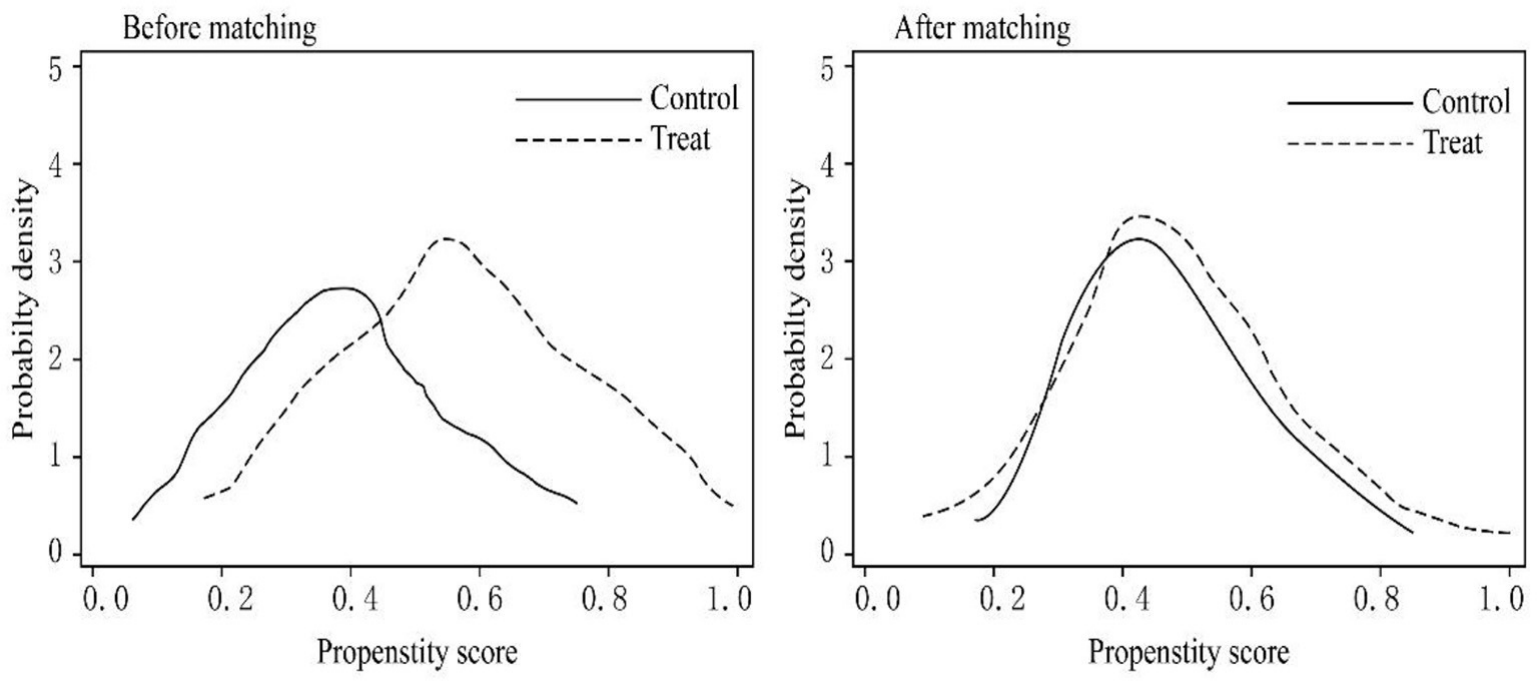
Common support domain of control and treat groups.

To ensure the quality of matching, we further discussed the common support area of the control group (Control) and the treatment group (Treat). We drew function density graphs before and after PSM (Figure 1). It is apparent from the propensity score values that matched vaccinated individuals and non-vaccinated individuals mostly overlapped, and the overlapping area is the common support area. Therefore, the data employed in the current research have better common support domain conditions; most of the observations are within the common value range. Additionally, in terms of the three different matching methods, the difference in sample loss is small. Table 3 shows the maximum loss of sample size. The treatment group lost 52 samples, the control group lost 246 samples, and 2,469 samples participated in the matching.

**Table 3:** Result of sample matching.

	**Vaccination equation**		
	**Unmatched sample**	**Matching sample**	**Total**
Control group	246	2,469	2,715
Treatment group	52	1,773	1,825
Total	296	4,242	4,540

#### Balance Test

After sample matching (Table 4), the overall standardization deviation of the covariate variables was <5%, significantly reducing the overall bias. In addition, the likelihood ratio (LR) value dropped significantly from 46.250 to 7.015–7.270, and the P–R^2^ value dropped from 0.615 to 0.024–0.027 after matching in the vaccination equation. The results show that PSM significantly reduces the covariate differences between the treatment and the control groups, and the sample matching quality is appropriate.

**Table 4:** Results of balance test.

**Matching method**	**Vaccination equation**		
	**P–R^**2**^**	**LR value**	**Standardization deviation**
Before sample matching	0.615	46.250	12.301
K-nearest neighbor matching	0.024	7.270	4.506
Caliper matching	0.027	7.015	4.302
Kernel matching	0.026	7.172	4.206

### The Effect of Vaccination Against COVID-19 on Participants' Health-Protective Measures

Table 5 shows the effect of vaccination against COVID-19 on participants' health-protective measures based on three different matching methods. Although various matching methods are applied, the direction and degree of the COVID-19 vaccination influencing participants' health-protective measures are the same, indicating that the estimated results have good robustness.

**Table 5:** The effect of the COVID-19 vaccination on participants' health-protective measures.

**Matching method**	**Health protective measures**	***ATT***	**Standard deviation**	***T***
K-nearest neighbor matching	Wearing mask	−0.102	0.066	1.54
	Handwashing	−1.749^***^	0.663	2.64
	Keeping physical distancing	−1.241^**^	0.577	2.15
Caliper matching	Wearing mask	−0.104	0.667	1.56
	Handwashing	−1.752^***^	0.656	2.67
	Keeping physical distancing	−1.238^**^	0.571	2.17
Kernel matching	Wearing mask	−0.102	0.066	1.55
	Handwashing	−1.750^**^	0.668	2.62
	Keeping physical distancing	−1.240^**^	0.574	2.16
Mean	Wearing mask	−0.103		
	Handwashing	−1.750		
	Keeping physical distancing	−1.240		

*, **, *** *Represent the significance level of 10, 5, and 1%, respectively*.

COVID-19 vaccination does not have a significant influence on participants' mask-wearing, indicating that, in China, regardless of whether people are vaccinated or not, they still choose to wear masks in public places, even in the post-epidemic era (69, 70). Consistent with the research by Liao et al. (71) and Ma et al. (72), our research also confirms that consistent mask-wearing behavior is contributing to the success of China in fighting the COVID-19 outbreak, and it provides a good example for other countries of how to cope effectively with the COVID-19 resurgence. Furthermore, we propose the possible reasons for mask-wearing as follows: First, regardless of the risk level of the COVID-19 epidemic and the degree of herd immunity realization, the Chinese government strictly implements a policy of wearing masks in public places, making mask-wearing a necessary condition for people accessing goods and services (73–75). Second, the epidemic experience is an important driving factor that affects people's behavioral changes. Unlike the traditional rational behavior theory, bounded rationality theory emphasizes forces other than individual welfare that influence behavior (76, 77). Wearing masks may affect people's subjective well-being, such as the perceived need to absorb the fresh air. However, the epidemic experience can make people pay more attention to health safety measures after their vaccination and consistently wear masks in public places (78, 79). Finally, as other scholars have emphasized, wearing masks may be limited by the cultural traditions of different countries (80, 81). If policy interventions are gradually relaxed, the probability of wearing masks will decrease. This situation is more likely to happen after vaccination (73). Consequently, given that herd immunity has not yet been formed, countries should take continuous measures to compel or motivate people to wear masks (82).

COVID-19 vaccination significantly decreases the number of times participants washed their hands by 1.75 per day. It is difficult for people to avoid being in an environment with hidden risks of SARS-CoV-2 infection, such as vegetable markets, supermarkets, and subway stations. No one knows whether an infected person has touched public facilities like railings, elevator buttons, and access switches. Therefore, washing hands frequently has been highly recommended by the WHO in the COVID-19 era. Two aspects can explain the reason for less frequent handwashing after vaccination.

On the one hand, Gharpure et al. (23) argued that, compared with the mask-wearing policy, it is difficult for the government to set out a handwashing policy and to set a minimum standard for handwashing per day. Therefore, handwashing is not a core part of government intervention measures. The number of times for handwashing depends on epidemic risk, living habits, and government messaging (22, 83). Contrarily, vaccination reduces the psychological fear of the risk of exposure to the virus. Studies have confirmed that vaccination can alleviate people's mental states of loneliness, fear, anxiety, and depression during infectious disease outbreaks, strengthening people's conscious performance of health-protective behaviors such as handwashing (84–87). Additionally, other scholars also confirmed that other public health supplies such as hand sanitizer provided by the government after large-scale vaccination have been gradually reduced, which also reduces the number of times people wash hands to some extent (88, 89).

COVID-19 vaccination significantly reduces participants' compliance intensity, reducing physical distancing of more than 1 meter by 1.24 times per day. In public places in China, red lines painted on the ground ensure that, when waiting in line, people comply with physical distancing generally of more than 1 meter. Related research by some scholars has shown that the COVID-19 outbreak extends people's physical and psychological distance (89, 90). The obstacles to implement the policy of maintaining physical distance are linked to the management and control of public health and of people's needs for close emotional communication (91). Studies have confirmed that the balance point for maintaining public welfare and emotional needs depends on the risk level of COVID-19 (92). Specifically, China has already controlled the epidemic well, and the quick roll-out of vaccination has caused people's risk awareness to decrease gradually. People are no longer limited by space restrictions and by the need for online communication. As a result, social activities have increased significantly (56, 58). Additionally, vaccination has reduced people's exposure to SARS-CoV-2, and the reduction in infections has encouraged their complacency to return to their pre-pandemic physical distancing (93).

As of April 2020, China had controlled the COVID-19 spread. Nevertheless, during the recovery process, there were clusters of COVID-19 cases, indicating a possible fall-off in the intensity of people's protective measures such as handwashing and maintaining physical distance leading to potential COVID-19 resurgence. Despite the current large-scale vaccination program in China, the protection period and effectiveness of the vaccine still require long-term scientific observation. Therefore, it is still necessary for the government to promote health-protective measures with the resumption of work and production.

### Research Limitations

Here, we outlined the limitations of our study. First, different vaccines have different efficacies, which calls for different strategies to combat unforeseen variants, such as Alpha, Beta, Gamma, and Delta variants (94). Currently, mRNA vaccines are considered the most protective vaccine with 90–100 efficacy (47). With the increased rate of vaccinations in the USA, the CDC has recommended that inoculated Americans can meet without wearing masks. Consequently, the research is not globally representative. Second, our research does not distinguish among mask-wearing for anti-COVID-19 or for air pollution. This public propensity for protection against air pollution such as smog may have conditioned them to continue wearing face masks. Consequently, the effect of vaccination against COVID-19 on wearing mask may be over-estimated. Third, ventilation is a primary control strategy for infectious diseases, which promotes the air dilution around a source and the removal of respiratory viruses (95). Recommendations have been introduced to reduce the transmission risk of virulent airborne viral particles by increasing ventilation rates, expressed in air-changes-per-hour (ACH), effectively improving the dilution of airborne pathogens via mechanical ventilation (96). However, limited to the original data acquisition, this research did not analyze the impact of the COVID-19 vaccination on ventilation measures. Finally, the PSM model is employed to analyze the net effect of vaccination against COVID-19 on participants' attitude toward protective countermeasures. However, the PSM model cannot simultaneously address the effects of other variables such as gender, age, education level, individual health risk perception, public health risk perception, social responsibility, peer effect, and government supervision on participants' health-protective measures. These shortcomings provide exciting avenues for future research.

## Conclusions and Implications

The tremendous damage caused by COVID-19 to global economic and social development is beyond statistical estimation. It is a matter of grave concern that SARS-CoV-2 traceability network is not yet in place. Human experience in combating infectious diseases shows that vaccines are the most fundamental measure. Unfortunately, the vaccine's protective efficacy, protection period, and the constant threat of variants challenge to the effectiveness of the COVID-19 vaccination. There is still a long and difficult path to the formation of worldwide herd immunity.

Consequently, vaccinated and non-vaccinated individuals should continue to engage in personal health-protective measures. This paper collected data from 4,540 individuals (1,825 vaccinated and 2,715 not vaccinated) in China and applied the PSM model to analyze the impact of vaccination against COVID-19 on participants' health-protective measures such as wearing masks, handwashing, and keeping physical distance to answer whether participants' protective measures against a resurgence of SARS-CoV-2 were weakened after their vaccination.

The main findings show that participants' gender, age, education level, individual health risk perception, public health risk perception, social responsibility, peer effect, and government supervision are the main factors affecting their vaccination choice. However, cultural roots and accessibility to health-protection products do not significantly influence participants' vaccination intention. Vaccination against COVID-19 significantly decreases participants' handwashing frequency by 1.75 times per day and reduces the compliance intensity of the observation of physical distancing of more than 1 meter by 1.24 times per day. Surprisingly, vaccination against COVID-19 does not have a significant influence on mask-wearing. Although China has controlled the COVID-19 outbreak well, people still choose to wear masks, providing a valuable example to other countries to successfully combat the epidemic. Of course, the compliance behavior model of mask-wearing may be strengthened by the COVID-19 experience, or due to culture, air pollution, and previous public health education impact. However, we should also accept that handwashing and keeping physical distance have gradually weakened, indicating that until herd immunity is achieved, China is still threatened by another outbreak of COVID-19.

Restoring economic activities around the world and strengthening people's health-protective measures are complementary rather than contradictory aims. The current research provides suggestions for policymakers to sustainably prevent and control COVID-19. First, the government should continually strengthen interventions related to people's health-protective measures. Specifically, the government should use multiple channels to promote the importance of frequent handwashing for reducing SARS-CoV-2 spread. In addition, the government should continue to strengthen the practice of physical distancing in public places to reduce the risk of human-to-human transmission of the virus. Second, the government should continue to increase the free supply of hand sanitizer, masks, in public places to reduce the cost to people of taking health-protective measures. Finally, the government should continue to trace the source and mutations of SARS-CoV-2, design and develop targeted vaccines, continuously improve the effectiveness of the COVID-19 vaccines, and finally achieve group immunity.

## Data Availability Statement

The raw data supporting the conclusions of this article will be made available by the authors, without undue reservation.

## Author Contributions

RS and YY contributed to the conception and design of the research. XZ and QL performed the statistical analysis. NA wrote sections of the manuscript. All authors participated in obtaining data, contributed to the article, and approved the submitted version.

## Conflict of Interest

The authors declare that the research was conducted in the absence of any commercial or financial relationships that could be construed as a potential conflict of interest.
